# Electrochemical Reduction and Preparation of Cu-Se Thermoelectric Thin Film in Solutions with PEG

**DOI:** 10.3390/nano12183169

**Published:** 2022-09-13

**Authors:** Yanling Qi, Yuanyuan Li, Wei Wang

**Affiliations:** Department of Applied Chemistry, School of Chemical Engineering and Technology, Tianjin University, Tianjin 300072, China

**Keywords:** Cu_2_Se, Seebeck coefficient, thermoelectric material, thin film

## Abstract

Investigation of Cu(II) and Se(IV) electrochemical reduction processes in solutions with poly(ethylene glycol) (PEG) provides important theoretical guidance for the preparation of Cu-Se alloy films with stronger thermoelectric properties. The results reveal that PEG adsorbing on the electrode surface does not affect the electrochemical reduction mechanism of Cu(II), Se(IV), and Cu(II)-Se(IV), but inhibits the electrochemical reduction rates. The surface morphology and composition change with a negative shift in the deposition potentials. The Cu-Se alloy film, which was prepared at 0.04 V, was α-Cu_2_Se as-deposited and P-type thermoelectric material after annealing. The highest thermoelectric properties were as follows: Seebeck coefficient (α) was +106 μV·K^−1^ and 1.89 times of Cu-Se alloy film electrodeposited in Cu(II)-Se(IV) binary solution without PEG; resistivity (ρ) was 2.12 × 10^−3^ Ω·cm, and the calculated power factor (PF) was 5.3 μW·cm^−1^K^−2^ and 4.07 times that without PEG.

## 1. Introduction

In the context of the rapid development of the global economy and the rapid increase in the world’s population, non-renewable resources are consumed in large quantities. Thermoelectric technology provides a new method of energy utilization and conversion and has been vigorously developed. At present, the application of thermoelectric technology is mainly in thermoelectric power generation and semiconductor refrigeration.

Cu_2_Se is one of the promising thermoelectric materials because of its intrinsically low thermal conductivity. For bulk Cu_2_Se prepared by spark plasma sintering, “the resistivity is on the order of 10^−2^~10^−3^ Ω·cm, and the Seebeck coefficient in the β-phase range of temperatures from 420 K to 1000 K varies between +80 and +300 μV/K. Based on the measured electrical resistivity and high Seebeck coefficient, the calculated power factor for the β-phase ranges from 7 to12 μW·cm^−1^K^−2^” [1].

There are many methods of preparing Cu_2_Se thin films [2,3,4,5,6,7,8,9,10]. For instance, Wang et al. [11] used pulsed laser deposition to prepare Cu_2_Se films. The in-plane directional power factor of the film is as high as ~20.02 μW/cm K^−2^ at 580 K and ~8.44 μW/cm K^−2^ at room temperature, which is higher than that of Cu_2_Se bulk. Zheng et al. [12] used a layer-by-layer composite reaction method to prepare high-performance copper selenide (Cu_2_Se) thin films. A high-power factor of 5.3 μWcm^−1^K^−2^ and a dimensionless quality factor of 0.35 were achieved at room temperature. The film has good flexibility, and the designed flexible device shows stable output power. Among these methods, electrochemical deposition has the advantages of low cost, simple process, and environmental protection. Tishkevich et al. [13] used a newly developed perchlorate electrolyte to electrodeposit Bi films. It has great application potential in the fields of sensing and radiation shielding. Although electrodeposited Cu_2_Se thin films are widely studied [14], the main applications of deposited Cu_2_Se thin films are focused on solar cells, and there are few reports on thermoelectricity.

In this report, the effects of PEG on electrochemical reduction processes of Cu(II) and Se(IV) were investigated by linear sweep voltammetry (LSV) and electrochemical impedance spectroscopy (EIS), and electrodeposited Cu-Se thin films in solution with PEG. The Cu-Se thin films had stronger thermoelectric properties than those deposited in solution without PEG. This observation presents a facile method for obtaining Cu-Se thin films with better thermoelectric performance.

## 2. Materials and Methods

Table 1 shows the solution composition: all reagents are analytical grade. Concentrated sulfuric acid was dissolved in secondary distilled water to obtain 0.050 M sulfuric acid solution, K_2_SO_4_ was dissolved in 0.050 M sulfuric acid solution, CuSO_4_∙5H_2_O and PEG-10000 were dissolved separately in the sulfuric acid solution with K_2_SO_4_, and a small amount of concentrated PEG solution was added into CuSO_4_ solution. H_2_SeO_3_ solutions were prepared in the same way.

All electrochemical measurements and electrodepositions were carried out using the three-electrode system, and with an electrochemical workstation (CHI660E, Shanghai Chenhua Instruments Co., Ltd., Shanghai, China). The working electrode was made of gold in the electrochemical measurements and of copper in the electrodepositions; the counter electrode was platinum titanium mesh, and the reference electrode was saturated calomel electrode (SCE). The measurement parameters of LSV were scan rate of 200 mV/s and potential range of open circuit potential of −1.25 V (vs. SCE). The measurement parameters of EIS were potential amplitude of 5 mV and frequency range of 10 mHz to 10 kHz. All the electrochemical measurements and electrodepositions were carried out at 25 ± 1 °C; the electrochemical measurements were conducted with no stirring, and the electrodepositions were conducted with magnetic stirring. The deposit capacity was 40 C. All potentials in this study were relative to SCE.

Surface morphology was obtained with a JEOL JSM-6700F (JEOL, Tokyo, Japan) scanning electron microscope (SEM); element composition was obtained with an electron dispersive spectroscopy (EDS), which was attached to the FEI Nanosem 430 (Thermo Electron Corporation, Waltham, USA); X-ray diffraction (XRD) pattern was obtained with a Rigaku D/max 2500 V/pc X-ray diffractometer (BRUKER AXS GMBH, Göttingen, Germany) using Cu Kα with a scan rate of 3°/min; the thickness of the deposited film with a microscope (Zeiss IMc5, Carl Zeiss AG, Oberkochen, Germany) was determined by calibrating the collected photos; Seebeck coefficient was measured with a Seebeck coefficient measurement system (TJU-EC5001) developed by Tianjin University, Tianjin, China; and resistivity was determined with a four-probe measurement system (TJU-EC1001) developed by Tianjin University at 18 ± 1 °C. The test principles of the Seebeck coefficient and resistivity are described in references [15,16]. The Cu-Se thin films to be tested in Seebeck coefficient and resistivity measurements were peeled off from copper substrates with high-temperature resistant epoxy adhesive and annealed at 250 °C for 6 h under N_2_ protection.

## 3. Results

### 3.1. Effect of PEG on the Electrochemical Reduction of Cu(II) and Se(IV)

#### 3.1.1. LSV Analysis

**Figure 1:**
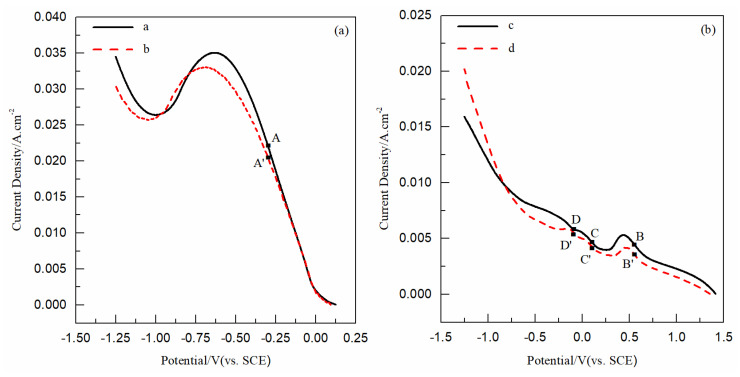
LSV curves obtained in Cu(II)/Se(IV) unitary solutions: (**a**) curve a in solution 1 without PEG, curve b in solution 2 with 4 μM PEG; and (**b**) curve c in solution 3 without PEG, curve d in solution 4 with 4 μM PEG. Scan rate: 200 mV/s.

Figure 1 shows the electrochemical reduction processes of Cu(II) and Se(IV) by LSV curves with solutions 1, 2, 3, and 4 in Table 1; (Cu(II) unitary solutions without and with PEG; and Se(IV) unitary solutions without and with PEG). Curve a and curve b in Figure 1a each have an obvious reduction peak, with about 0.00 V initial potentials, −0.63 V, and −0.69 V peak potentials. This corresponds to the reduction reaction of Cu^2+^ to Cu^0^:

(1) $$Cu^{2+}+2e^{-}\to Cu^{0}$$

There was little effect on the initial potentials and peak shape of the curve a and b, indicating that the addition of PEG does not affect the reduction mechanism of Cu(II). From the peak potential shifting 0.06 V negatively and the reduction current decreasing slightly as a whole, it can be inferred that the addition of PEG inhibits the reduction rate of Cu^2+^ on the electrode surface.

Curve c and curve d in Figure 1 ball has three reduction peaks: the first reduction peaks with about 0.70 V initial potentials, and 0.43 V and 0.47 V peak potentials; the second reduction peaks with about 0.25 V initial potentials, and −0.04 V and 0.01 V peak potentials; the third reduction peaks with about −0.06 V and 0.00 V initial potentials, and −0.35 V and −0.14 V peak potentials. These three reduction peaks correspond to reduction reactions of H_2_SeO_3_ to H_2_Se [17]. The equations are as follows:

(2) $$H_{2}SeO_{3}+4H^{+}+2e^{-}\to Se^{2+}+3H_{2}O$$

(3) $$Se^{2+}+2e^{-}\to Se^{0}$$

(4) $$Se^{0}+2H^{+}+2e^{-}\to H_{2}Se\left(aq.\right)$$

However, in fact, there was no evolution of gaseous H_2_Se on the electrode. According to a previous research report [18], in the presence of H_2_SeO_3_, H_2_Se in acid solution is unstable and will generate Se through chemical reaction Equation (5).

(5) $${2H}_{2}\mathrm{Se}\left(\mathrm{aq}.\right){+H}_{2}{\mathrm{SeO}}_{3}\to {3\mathrm{Se}+3H}_{2}O$$

There was little effect on the initial potentials and peak shapes of curve a and b, indicating that the addition of PEG has no effect on the electrochemical reduction mechanism of Se(IV). It can be inferred that the addition of PEG inhibits the reduction rate of H_2_SeO_3_ on the electrode surface, indicated by the peak potential shifting and the reduction current decreasing in the potential range of 0.8 V~−0.8 V (i.e., within the potential range of three reduction peak).

#### 3.1.2. EIS Analysis

**Figure 2:**
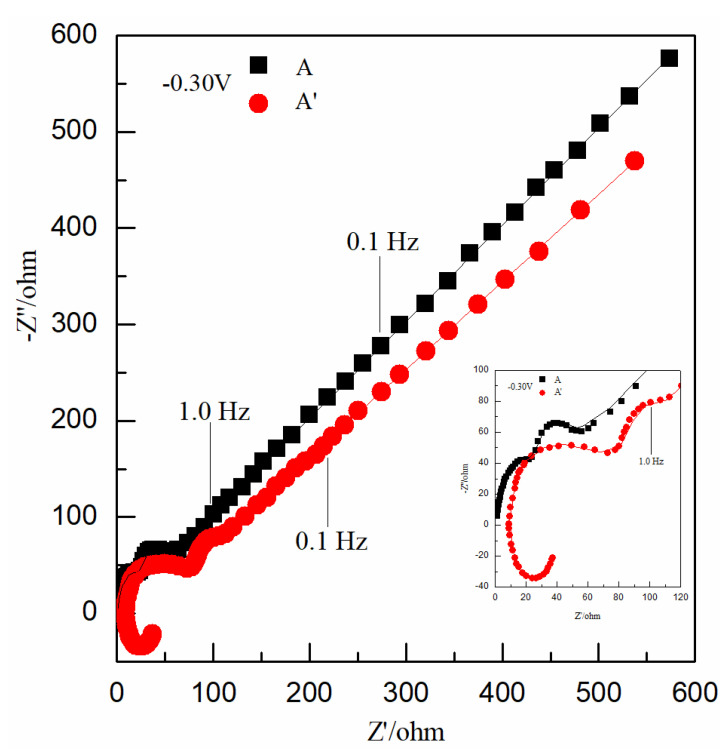
Nyquist plots obtained at −0.30 V in Cu(II) unitary solutions: curve A in solution 1 without PEG; curve A′ in solution 2 with 4 μM PEG. Inset: Partly amplified Nyquist plots of Figure 2.

Figure 2 shows Nyquist plots of points A and A′ (−0.30 V), which were selected in the reduction potential ranges of Figure 1a. Curve A in Figure 2 consists of two semicircles and a line; curve A’ has one more inductive loop than curve A. The inductive loop suggests that there was an adsorption reaction of PEG on the electrode surface, and the frequency of the inductive loop was higher than that of two semicircles, indicating that the adsorption reaction of PEG on the Au electrode surface occurred before the reduction of Cu(II); the two semicircles suggest that the electrochemical reduction reaction of Cu^2+^ to Cu^0^ was carried out by two reactions and one electron for each reaction. The equations are as follows [19]:

(6) $${\mathrm{Cu}}^{2+}+e^{-}\to {\mathrm{Cu}}^{+}$$

(7) $${\mathrm{Cu}}^{+}+e^{-}\to {\mathrm{Cu}}^{0}$$

The two lines of curve A and A′ in Figure 2 are basically parallel with a slope of about 45°, indicating that the low-frequency ranges of curve A and A′ are controlled by concentration diffusion. The results of curve A and A′ in Figure 2 show that the adsorption of PEG on the Au electrode surface had no effect on the reduction mechanism of Cu(II).

**Figure 3:**
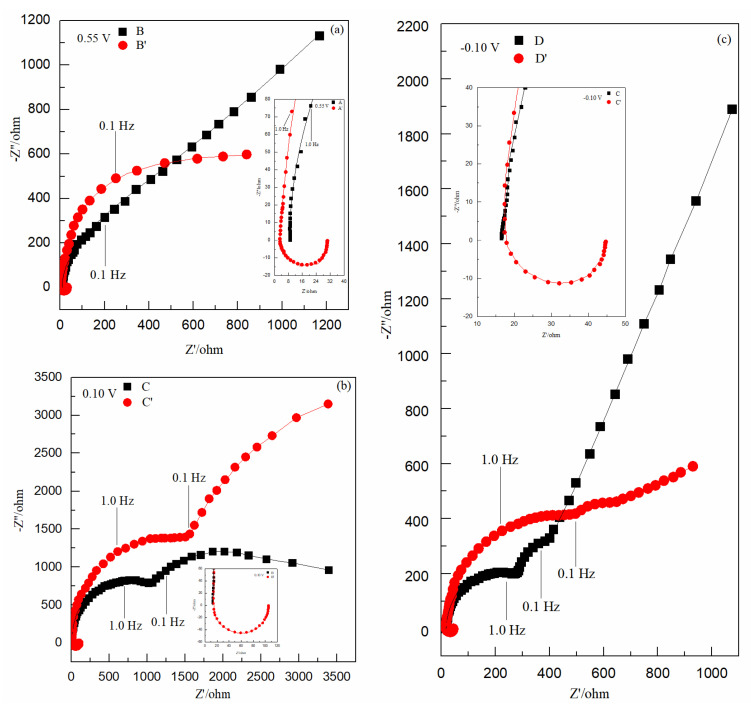
Nyquist plots obtained at different potentials in Se(IV) unitary solutions: (**a**) 0.55 V, curve B in solution 3 without PEG; curve B′ in solution 4 with 4 μM PEG; (**b**) 0.10 V, curve C in solution 3 without PEG; curve C′ in solution 4 with 4 μM PEG; and (**c**) −0.10 V, curve D in solution 3 without PEG; curve D′ in solution 4 with 4 μM PEG. Inset: Partly amplified Nyquist plots of (**a**–**c**).

Figure 3 shows Nyquist plots of points B and B′ (−0.30 V), point C and C′ (0.10 V), and points D and D′ (−0.10 V), which were selected in the three reduction potential ranges of Figure 1b.

Curve B′, curve C′, and curve D′ in Figure 3 all have one more inductive loop than curve B, curve C, and curve D; these inductive loops suggest that there was an adsorption reaction of PEG on the electrode surface, and the frequency of the inductive loops were all higher than that of the semicircles, indicating that the adsorption reaction of PEG on Au electrode surface occurred before the reduction of Se(IV).

Curve B and B′ in Figure 3a all have one semicircle and a line: one semicircle suggests that the electrochemical reduction reaction of H_2_SeO_3_ to Se^2+^ was carried out by one electrochemical reaction that obtained two electrons, as shown in Equation (2). The slope of the straight-line part of curve B is about 45°, and the slope of the short straight-line part of curve B′ is less than 45°, indicating that the low-frequency ranges of curve B and curve B′ were controlled by concentration diffusion and activation.

Curve C and C′ in Figure 3 ball have two semicircles: the two semicircles suggest that the electrochemical reduction reaction of Se^2+^ to Se^0^ was carried out by two electrochemical reactions and one electron for each reaction. The Equations are as follows:

(8) $${\mathrm{Se}}^{2+}+e^{-}\to {\mathrm{Se}}^{+}$$

(9) $${\mathrm{Se}}^{+}+e^{-}\to {\mathrm{Se}}^{0}$$

Curve D and D′ in Figure 3c all have two semicircles and a line: the two semicircles suggest that the electrochemical reduction reaction of Se^0^ to H_2_Se was carried out by two electrochemical reactions and one electron for each reaction. The possible equations are as follows:

(10) $${\mathrm{Se}}^{0}{+H}^{+}+e^{-}\to \mathrm{HSe}(\mathrm{aq}.)$$

(11) $$HSe\left(aq.\right)+H^{+}+e^{-}\to H_{2}Se\left(aq.\right)$$

The slope of the straight-line part of curve D is about 45°, and the slope of the short straight-line part of curve D′ is less than 45°, indicating that the low-frequency ranges of curve D and curve D′ were controlled by concentration diffusion and activation. The results in Figure 3 show that the adsorption of PEG on the Au electrode surface did not affect the electrochemical reduction mechanism of Se(IV).

**Figure 4:**
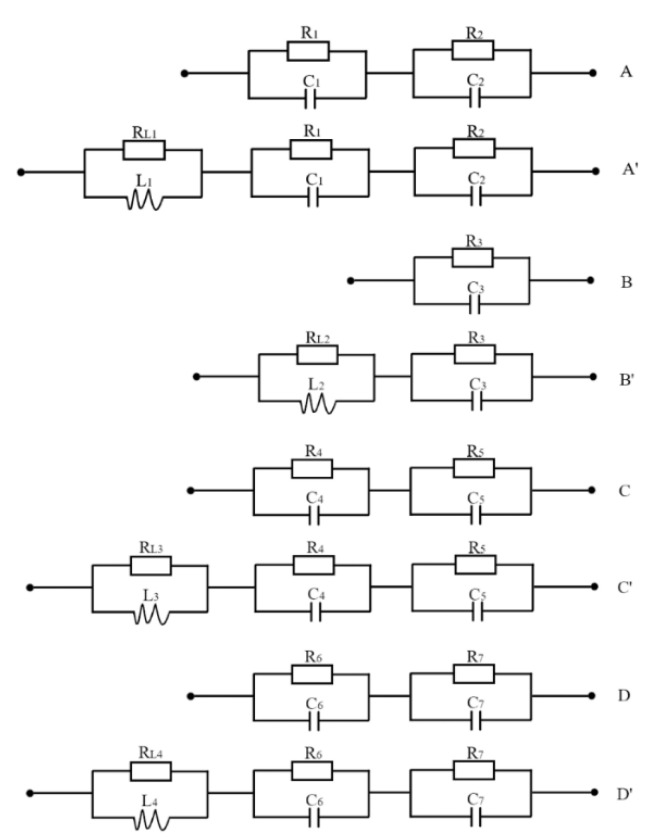
Equivalent circuits of the EIS in Figure 2 and Figure 3. Circuits A, A′, B, B′, C, C′, D, and D′ correspond to curves A, A′, B, B′, C, C′, D, and D′ in Figure 2 and Figure 3. R_1_, R_2_, R_3_, R_4_, R_5_, R_6,_ and R_7_, C_1_, C_2_, C_3_, C_4_, C_5_, C_6,_ and C_7_: the electrochemical reaction resistances and double layer capacitances of Equations (2), (6)–(11); L_1_, L_2_, L_3_ and L_4_, R_L1_, R_L2_, R_L3,_ and R_L4_: the inductance and resistance caused by adsorption and desorption of PEG.

Figure 4 shows equivalent circuits of the Nyquist plots in Figure 2 and Figure 3. The simulated solid lines, which fit well with the Nyquist plots, are shown in Figure 2 and Figure 3. Table 2 shows some parameters of the simulated equivalent circuits. R_1_ was greater than R_2_ whether PEG was added or not, but the difference was small, indicating that the electron transfer process of Equation (6) was slightly more difficult than that of Equation (7). When PEG was added to the solution, R_1_ and R_2_ all increased, which shows that the adsorption of PEG on the Au electrode surface inhibited the reduction rate of Cu(II). R_5_ was greater than R_4_ whether PEG was added or not, indicating that the electron transfer process of Equation (9) was more difficult than that of Equation (8); R_6_ was greater than R_7_ whether PEG was added or not, indicating that the electron transfer process of Equation (10) was more difficult than that of Equation (11); R_4_ and R_5_ were all greater than R_3_, R_6,_ and R_7_, indicating that the electron transfer process of Equations (8) and (9) was more difficult than that of Equations (2), (10) and (11), i.e., the electrochemical reduction process of Se^2+^ to Se^0^ was more difficult than that of H_2_SeO_3_ to Se^2+^ and Se^0^ to H_2_Se, whether PEG was added or not. When PEG was added to the solution, R_3_, R_4_, R_5_, R_6,_ and R_7_ all increased, which shows that the adsorption of PEG on the Au electrode surface inhibited the reduction rate of Se(IV). PEG is a surfactant that can be adsorbed on the electrode surface. The adsorbed PEG covered the electrode surface and prevented the deposition of cations. On the macroscopic level, the reaction rate was reduced. This increased the resistance that represented the reaction rate in the AC impedance test [20,21]. Electric double-layer capacitance is a physical quantity related to the distance between the plates. The specific adsorption of PEG on the electrode surface reduced the thickness of the dense layer on the electrode surface. This increased the electric double-layer capacitance.

### 3.2. Effect of PEG on the Electrochemical Reduction of Cu(II)-Se(IV) 

**Figure 5:**
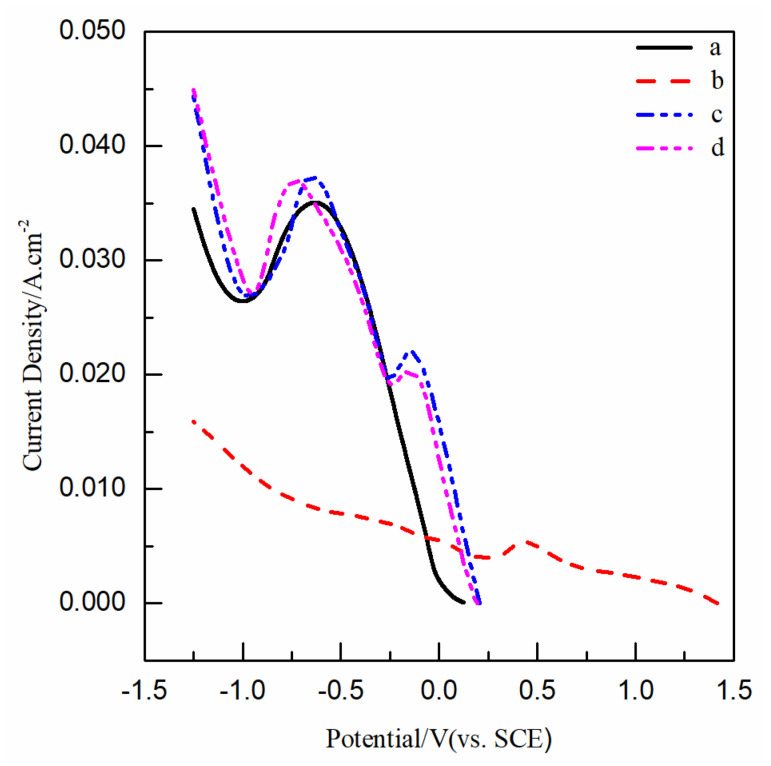
LSV curves obtained in different solutions. Curve a in solution 1 without PEG; curve b in solution 3 without PEG; curve c in solution 5 without PEG; and curve d in solution 6 with 4 μM PEG. Scan rate: 200 mV/s.

Figure 5 shows the electrochemical reduction processes of Cu(II)-Se(IV) by LSV curves with solutions 5 and 6 in Table 1 (Cu(II)-Se(IV) binary solutions without and with PEG). Curve c shows two reduction peaks; the first peak with 0.20 V initial potential and −0.15 V peak potential; curve d shows two reduction peaks; the first peak with 0.19 V initial potential and −0.16 V peak potential. Compared with the initial potentials around a range of 0.00 V~0.30 V in Figure 5, the initial potential of Se^2+^ in the solution without PEG is largest, Cu^2+^ without PEG is smallest, Cu(II)-Se(IV) without PEG and Cu(II)-Se(IV) with PEG is in the middle, and Cu(II)-Se(IV) without PEG is greater than Cu(II)-Se(IV) with PEG. This indicates that Se^2+^ is most easily reduced and Cu^2+^ is most difficultly reduced. Compared with the reduction currents of the curves a, b, and c in Figure 5, the current of Cu(II)-Se(IV) was higher than that of Cu^2+^ or H_2_SeO_3_ on the whole, which indicates that the reduction reaction in the Cu(II)-Se(IV) binary solution was the co-reduction reaction of Cu^2+^ and H_2_SeO_3_. Combined with the comparison of initial potentials and reduction currents, it can be inferred that Se^0^ induced the reduction of Cu^2+^ during the reduction process in Cu(II)-Se(IV) binary solution; that is, the co-reduction reaction of Cu(II) and Se(IV) was due to induced co-deposition. Compared with the reduction currents of curves c and d in Figure 5, the reduction current of Cu(II)-Se(IV) with PEG was slightly lower than that without PEG, but the shape of the current curves was basically the same; it can be inferred that the addition of PEG did not affect the induced co-deposition mechanism of Cu(II)-Se(IV), but inhibited the reduction rate of Cu(II)-Se(IV) on the Au electrode surface.

To prepare Cu-Se alloy thin film, it is necessary to select the potential suitable for Cu(II)-Se(IV) co-deposition based on comprehensive consideration of electrochemical co-deposition conditions, namely, the co-deposition potential within the potential range of Cu(II)-Se(IV) co-deposition; no reduction reaction of Se^0^ to H_2_Se; higher electrodeposition speed; and avoidance of the influence of ion diffusion in solution. Therefore, the co-deposition potential of Cu(II)-Se(IV) should be in the range of 0.19 V~−0.04 V (lower than the initial potential of Cu(II)-Se(IV) first reduction peak, higher than the maximum peak potential of Cu^2+^, Se^2+,^ and Cu(II)-Se(IV) reduction peaks). 

### 3.3. Effect of Deposition Potentials on the Surface Morphology and Composition of Cu-Se Thin Films

**Figure 6:**
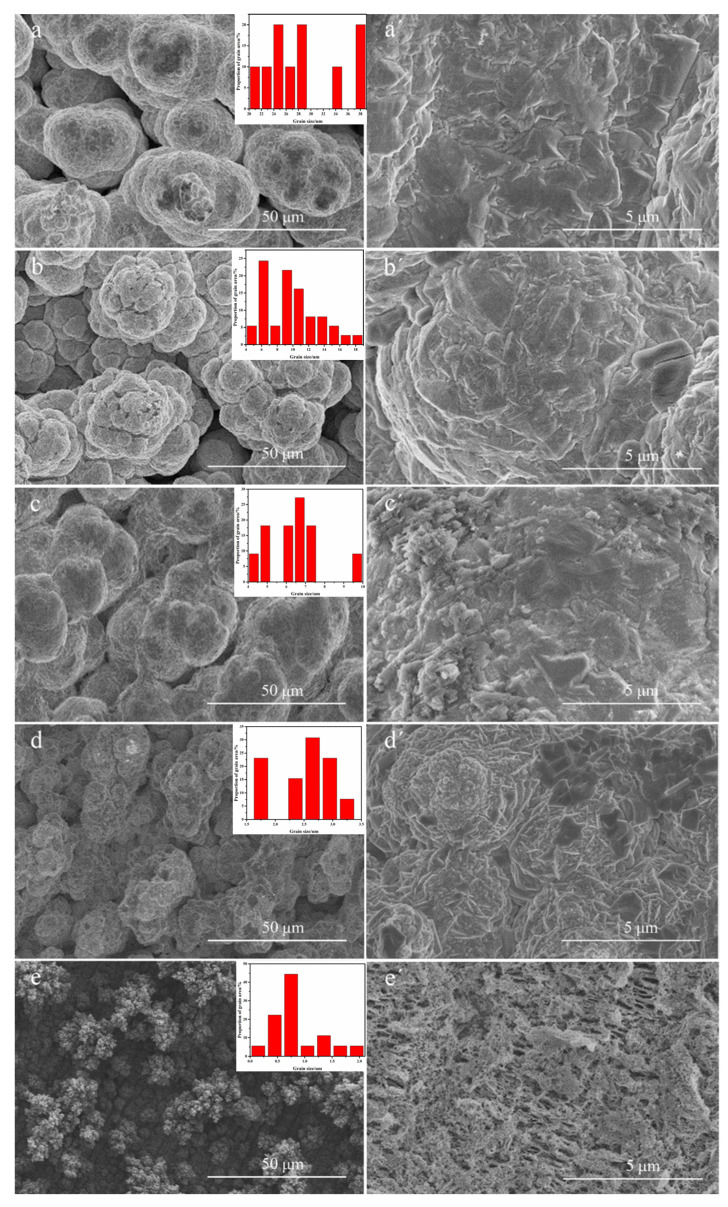
SEM images of Cu-Se film deposited at different potentials: (**a**–**e**) 0.065 V, 0.055 V, 0.040 V, 0.020 V, and 0.010 V, at 1000 magnification (Inserts: corresponding grain size distribution); and (**a′**–**e′**) 0.065 V, 0.055 V, 0.040 V, 0.020 V, and 0.010 V, at 10,000 magnification.

The SEM images of Cu-Se thin films electrodeposited in Cu(II)-Se(IV) binary solution with PEG (solution 6 in Table 1) at different deposition potentials are shown in Figure 6. The surface of the Cu-Se film deposited at 0.065 V was light gray and granular with uniform and large particle size; the space between particles was large, and the particle surface was relatively flat. The surface of the Cu-Se film deposited at a potential range of 0.055 V to 0.04 V was gray and granular with uniform and smaller particle size; the particles were gradually connected, and the particle surface was also relatively flat. The surface of Cu-Se film deposited at 0.02 V was gray black and rough, and the particle surface attained a cauliflower formation. The surface of the Cu-Se film deposited at 0.01 V was black with almost no particles and with a porous sponge formation. The average grain sizes of films were 28.25 μm, 9.77 μm, 6.41 μm, 2.54 μm, and 0.86 μm [22].

Compared with the surface morphology of Cu-Se films prepared at different potentials, the surface morphology became visibly worse when the deposition potential shifted negatively to 0.02 V. This was because, with the negative shift in the potential, the deposition current density was larger, resulting in the formation of more crystal nuclei, resulting in smaller grain size. As a result, the surface of the prepared material became rough.

**Figure 7:**
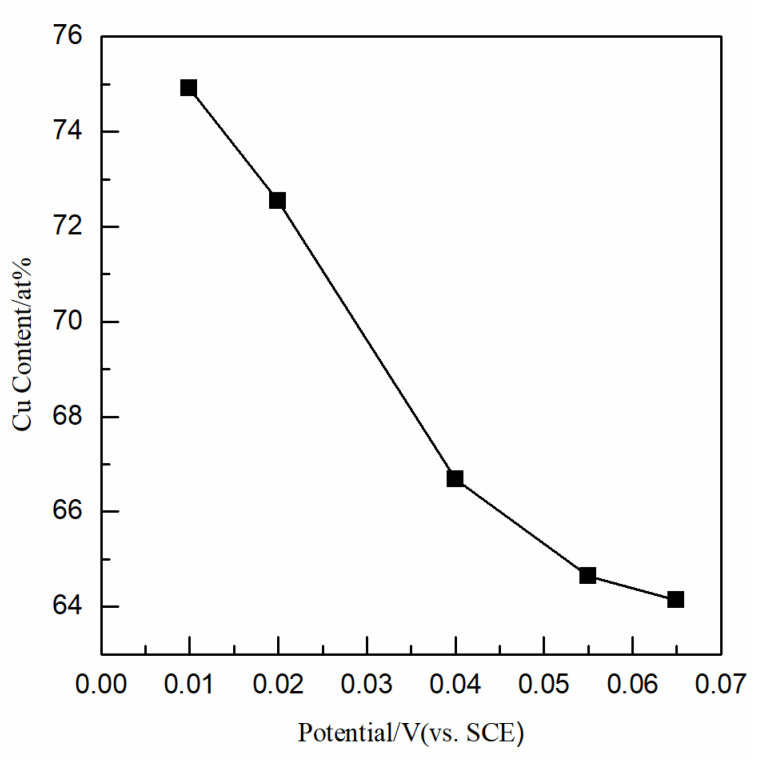
Variation of copper contents in Cu-Se film with deposition potentials.

Figure 7 shows the Cu contents of the Cu-Se films electrodeposited at different potentials. The Cu contents increased slowly from 64.14 at% to 66.68 at% with a negative shift in the deposition potentials from 0.065 V to 0.04 V; the Cu contents increased linearly from 66.68 at% to 74.91 at% with a negative shift in the deposition potentials from 0.04 V to 0.01 V. The Cu contents in the Cu-Se films increased with the negative shift in deposition potentials; this may be because with the greater negative deposition potential of Cu^2+^ than H_2_SeO_3_, more Cu^2+^ ions were reduced when the potential shifted negatively. 

Considering the effect of different deposition potentials on the surface morphology and composition of the Cu-Se films, the optimal deposition potential was 0.04 V.

## 4. Discussion

**Figure 8:**
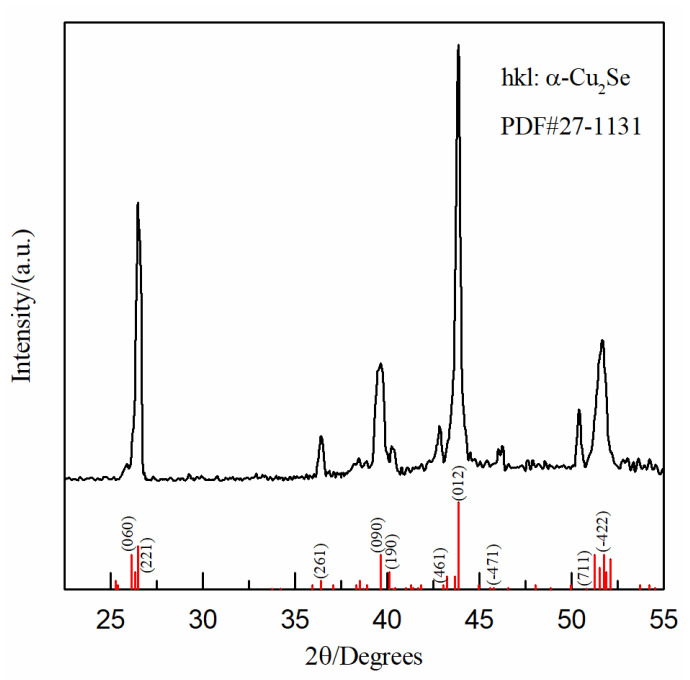
XRD pattern of Cu-Se film as-deposited.

The XRD pattern of the Cu-Se film (deposited at the potential of 0.04 V in Figure 7) is shown in Figure 8. The XRD pattern corresponded well with the standard diffraction peak of α-Cu_2_Se (PDF#27-1131), indicating that the Cu-Se film was α-Cu_2_Se.

The Cu-Se thin films were peeled off from copper substrate with high-temperature resistant epoxy adhesive, and thermoelectric properties were analyzed after annealing. The results show that the Seebeck coefficient α of the Cu-Se films was up to +106 μV·K^−1^, indicating the film was a *p*-type semiconductor, and the resistivity ρ was 2.12 × 10^−3^ Ω·cm. According to Equation (12), the power factor PF of the thin film was calculated as 5.3 μW·cm^−1^K^−2^.

(12) $${\mathrm{PF}=\alpha }^{2}/\rho$$

As previously reported by our research group, the highest thermoelectric properties of the Cu-Se film deposited in the solution without PEG were as follows [23]: α is +56 μV·K^−1^, and PF was 1.3 μW·cm^−1^K^−2^. It can be seen that α of the film deposited in the Cu(II)-Se(IV) binary solution with PEG was 1.89 times that without PEG, and PF was 4.07 times that without PEG. The reason may be that the addition of PEG increased the electrochemical reaction resistance of Cu(II)-Se(IV), inhibited the reduction rate of Cu(II)-Se(IV), refined the alloy grain, and made the electrodeposited film more compact, thus increasing α and reducing ρ, and finally increasing PF.

## 5. Conclusions

In this paper, the effects of PEG on the electrochemical reduction of Cu(II) and Se(IV) were investigated by LSV and EIS. PEG was able to adsorb on the Au electrode surface, which did not affect the electrochemical reduction mechanism of Cu(II) and Se(IV), but increased the electrochemical reaction resistance of Cu(II) and Se(IV), thus inhibiting the reduction rate of Cu^2+^ and H_2_SeO_3_ on the electrode surface; the addition of PEG did not affect the induced co-deposition mechanism of Cu(II)-Se(IV), but inhibited the deposition rate of Cu(II)-Se(IV). The co-deposition potential of Cu(II)-Se(IV) should be in the range of 0.19 V~−0.04 V.

Based on the study of electrochemical reduction processes, Cu-Se films were prepared, and the effects of deposition potentials on the surface morphology and composition were studied. The surface morphology of films began to deteriorate obviously with the deposition potential shifting from 0.065 V to 0.02 V, and the surface morphology was a porous sponge with the deposition potential of 0.01 V. The Cu content of the Cu-Se film increased from 64.14 at% to 74.91 at%, with the deposition potential shifting from 0.065 V to 0.01 V. According to the surface morphology and composition results, the optimal deposition potential of Cu-Se film was 0.04 V.

The Cu-Se film deposited at 0.04 V was α-Cu_2_Se. The thermoelectric properties of the annealed Cu-Se films were analyzed, and *p*-type thermoelectric materials were obtained. The highest thermoelectric properties were as follows: Seebeck coefficient α was +106 μV·K^−1^, resistivity ρ was 2.12 × 10^−3^ Ω·cm, and the calculated power factor PF was 5.3 μW·cm^−1^K^−2^.

This research shows that, for Cu-Se alloy, which can be co-deposited by itself, stronger thermoelectric properties could be obtained by adding surfactants or other reagents that could refine alloy grains in the solution.

## Figures and Tables

**Table 1:** Solution composition of electrochemical measurement and electrodeposition.

Solution	CuSO_4_·5H_2_O (M)	H_2_SeO_3_ (M)	PEG-10000 (μM)	K_2_SO_4_ (M)	H_2_SO_4_ (M)
1	0.070	-	-	0.140	0.050
2	0.070	-	4.000	0.140	0.050
3	-	0.045	-	0.140	0.050
4	-	0.045	4.000	0.140	0.050
5	0.070	0.045	-	0.140	0.050
6	0.070	0.045	4.000	0.140	0.050

**Table 2:** Related parameters in Figure 4.

Parameters	Without PEG	With 4 μM PEG
R_1_ (Ohm·cm^2^)	16.8	40.9
R_2_ (Ohm·cm^2^)	9.1	26.0
R_3_ (Ohm·cm^2^)	358.7	634.3
R_4_ (Ohm·cm^2^)	827.5	1210.7
R_5_ (Ohm·cm^2^)	1354.0	3362.6
R_6_ (Ohm·cm^2^)	200.0	408.9
R_7_ (Ohm·cm^2^)	127.2	200.4
C_1_ (F/cm^2^)	2.045 × 10^−7^	2.608 × 10^−7^
C_2_ (F/cm^2^)	6.198 × 10^−7^	8.293 × 10^−7^
C_3_ (F/cm^2^)	1.006 × 10^−4^	2.807 × 10^−4^
C_4_ (F/cm^2^)	7.108 × 10^10^	1.114 × 10^11^
C_5_ (F/cm^2^)	1.722 × 10^11^	3.012 × 10^11^
C_6_ (F/cm^2^)	2.337 × 10^−6^	3.467 × 10^−6^
C_7_ (F/cm^2^)	6.897 × 10^−6^	1.677 × 10^−5^
L_1_ (Henri/cm^2^)	-	4.76 × 10^3^
L_2_ (Henri/cm^2^)	-	1.85 × 10^3^
L_3_ (Henri/cm^2^)	-	6.48 × 10^5^
L_4_ (Henri/cm^2^)	-	4.71 × 10^3^

## Data Availability

Not applicable.
